# Profiles of immune infiltration and its relevance to survival outcome in meningiomas

**DOI:** 10.1042/BSR20200538

**Published:** 2020-05-14

**Authors:** Xiaodong Chen, Fen Tian, Peng Lun, Yugong Feng

**Affiliations:** 1 Department of Neurosurgery, The Affiliated Hospital of Qingdao University, 266003, Qingdao, Shandong, P.R.C.; 2 Department of Nephrology, The Affiliated Hospital of Qingdao University, 266003, Qingdao, Shandong, P.R.C.

**Keywords:** immune infiltration, meningiomas, prognosis, proportion

## Abstract

Tumor-infiltrating immune cells play a decisive part in prognosis and survival. Until now, previous researches have not made clear about the diversity of cell types involved in the immune response. The objective of this work was to confirm the composition of tumor-infiltrating immune cells and their correlation with prognosis in meningiomas based on a metagene approach (known as CIBERSORT) and online databases. A total of 22 tumor-infiltrating immune cells were detected to determine the relationship between the immune infiltration pattern and survival. The proportion of M2 macrophages was more abundant in 68 samples, reaching more than 36%. Univariate Cox regression analysis displayed that the proportion of dendritic cells was obviously related to prognosis. Hierarchical clustering analysis identified two clusters by the method of within sum of squares errors, which exhibited different infiltrating immune cell composition and survival. To summarize, our results indicated that proportions of tumor-infiltrating immune cells as well as cluster patterns were associated with the prognosis, which offered clinical significance for research of meningiomas.

## Introduction

Meningiomas are tumors that arise from the meninges of the brain and the spinal cord [1]. The incidence rate of meningioma is approximately 20%, making them the most common primary intracranial tumors in adults [2]. More than 90% of meningiomas are considered benign. A small proportion exhibit aggressive behavior with invasive growth patterns and higher rates of recurrence [3]. Multiple meningiomas are occasionally visible, with a family history reported in the literature. About 50% of them are located to the sagittal sinus and the other are located on the convex side of the brain and the falx side of the brain. Few of them are located in the ventricles of the brain, also seen in the epidural, but occasionally seen elsewhere. The standard treatment of grade II and grade III meningiomas involve surgery, radiation therapy and chemotherapy [4]. Despite the treatment efforts, the evolution of aggressive meningiomas remains unsatisfactory due to not only the high rates of local recurrence and/or tumor progression [5], but also insufficient understanding of the disease. Hence, understanding of mechanism in tumor progression is the mainly way to increase the survival rate of patients with meningiomas.

It has been well known that the malignant degree of tumor is not only defined by the intrinsic activity of tumor, but also relevant to the enrichment and activation of infiltrating immune cells in the microenvironment surrounding the tumor cells [6,7]. The composition and function of tumor-infiltrating immune cells make changes according to the immune status of the host [8]. Many researches have demonstrated that tumor-infiltrating immune cells could remarkably respond to certain targeted drugs and are correlated with clinical outcomes [9,10]. The research on mechanism have proved that tumor-infiltrating immune cells plays a dual role in promoting or inhibiting tumor, and thus discovering the complicated relationship between infiltrating immune cells and tumor cells [11,12]. Due to the technical constraints, most previous studies used immunohistochemistry or flow cytometry to evaluate the composition of immune infiltrating cells in tumors, ordinarily including only one or two cell types [13,14]. For the sake of better investigating the composition and role of the immune cells in cancer, a more precise approach is needed.

We used CIBERSORT – a method to evaluate immune infiltrating cells subpopulations using a deconvolution algorithm based on gene expression data [15,16], to reveal the composition and proportion of tumor-infiltrating immune cells and explore the relationship between tumor-infiltrating immune cells as well as immune cluster patterns and prognosis. We speculated that this work provided a great exploratory significance in clinical research.

## Materials and methods

### Data sources

The data in the present study were obtained from the gene expression matrices of meningiomas numbered GSE16581 in the GEO dataset. There were 68 samples of GEO dataset, all of which were cancer samples, and 67 samples had complete clinical survival information.

### Data processing

#### Calculation of tumor-infiltrating immune cells

We calculated the relative proportions and *P*-value of 22 kinds of infiltrating immune cells in each sample by CIBERSORT software [17,18]. The CIBERSORT software was applied to characterize the composition of infiltrating immune cells by deconvolution algorithm to use the preset 547 barcode genes based on a gene expression matrix. Here, gene expression matrix is the normalized mRNA levels in the microarray across all the detected samples. The 547 barcode genes compose a signature matrix which named LM22 that was initially primarily used in gene microarray dataset for reliable differentiation of 22 mature human hematopoietic populations, while its applicability in RNA-seq dataset was also recently confirmed.

Furthermore, the immune cell lytic activity of infiltrating immune cells was obtained by calculating the average expression levels of genes GZMA and PRF1, which was also a method for understanding the composition of infiltrating immune cells [19–21].

#### Enrichment analysis of GSEA gene set

According to the content of immune infiltrating cells, the samples were divided into groups. All samples were selected, and a gene set enrichment analysis were performed for all the genes by GSEA3.0 software in the samples [22,23]. The selected gene set was Kyoto Encyclopedia of Genes and Genomes (KEGG) pathway. *P*<0.05 was set as the threshold for screening markedly enriched KEGG pathways.

#### Statistical analysis

For 67 samples with survival information, the survival analysis was carried out in two groups (*P*<0.05 and *P*>0.05) by using the survival package. At the same time, the relative content of immune infiltrating cells was divided into high and low groups according to the median. The Cox risk ratio regression model was used to calculate the risk ratio (HR), and the survival analysis was carried out for the significant influencing factors [24,25]. Finally, we used the Euclidean distance method to hierarchically cluster 68 samples, and calculated the composition of immune infiltrating cell composition in each class of sample, respectively, and display the survival time curve of each class. Statistical analyses were performed using SPSS or GraphPad Prism software.

## Results

### The performance of CIBERSORT

**Figure 1:**
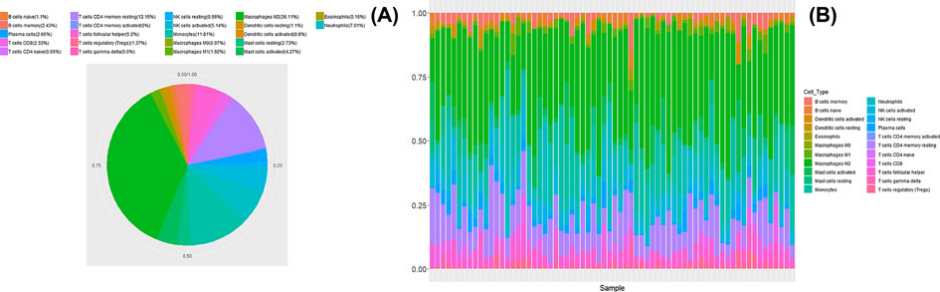
Patterns of tumor-infiltrating immune cells in meningiomas (**A**) The proportions of tumor-infiltrating immune cells in 68 meningiomas samples from GEO dataset. (**B**) The composition of 22 types of tumor-immune infiltration cells in 68 meningiomas samples from GEO dataset.

Using CIBERSORT software, we successfully identified human leukocyte subpopulations in meningiomas. As shown in Figure 1A, we could see the proportion of different immune cells in the samples from the GEO dataset. It could be observed that these samples contain more M2 macrophages cells, reaching more than 36%.

### Composition of tumor-infiltration immune cells in meningiomas

Using the CIBERSORT algorithm, we investigated the differences of immune infiltration among tumor tissues in 22 immune cell subsets. Figure 1B summarized the composition of immune infiltrating cells in tumor samples. It could be seen from the figure that the composition of immune cells was very different. Therefore, we could speculate that the change in the proportion of tumor-infiltrating immune cells might be an inherent feature, which could characterize individual and disease differences.

### The *P*-value of CIBERSORT reflected the overall proportion of immune cells

**Figure 2:**
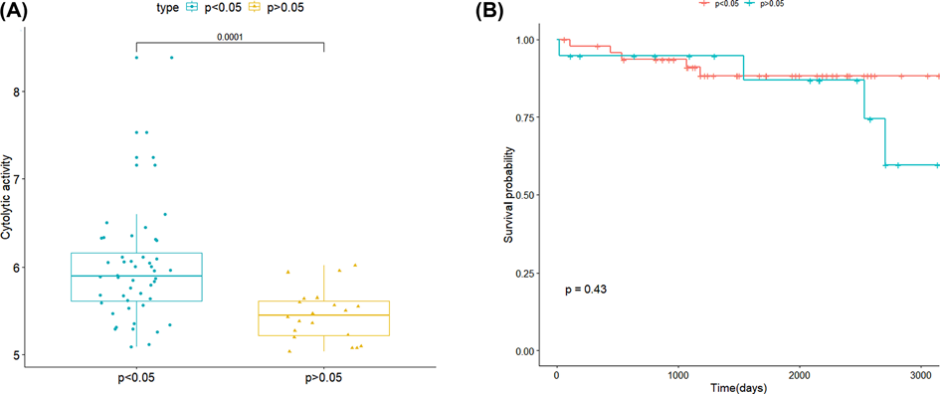
The association between CIBERSORT *P*-value and the composition of tumor-infiltrating immune cells (**A**) The association between immune cytolytic activity and CIBERSORT *P*-value. The proportion of tumor-infiltrating immune cells with *P*>0.05 and *P*<0.05 in 68 meningiomas samples from GEO dataset. (**B**) The Kaplan–Meier survival curve in meningiomas 67 samples from GEO dataset

The CIBERSORT algorithm only provided information about the relative proportion between subpopulations, not actual values, which made the results not independent of each other. Therefore, we studied the relationship between the *P*-value size of all samples and the composition of immune infiltrating cells. We used the average value of the genes GZMA and PRF1 to define the lytic activity of immune infiltrating cells. The larger the value and the stronger the cytolytic activity, which suggested the larger the proportion of immune infiltrating cells. It could be seen in Figure 2A that the cytolytic activity of the sample with *P*<0.05 was significantly higher than that of the sample with *P*>0.05, indicating that the proportion of immune infiltrating cells of the sample with *P*<0.05 was significantly higher than that of the sample with *P*>0.05.

A further study of the *P-*value and prognosis of 67 samples with survival information found that the *P-*value did not significantly affect the prognosis of survival (*P*>0.05), as shown in Figure 2B for details. Accordingly, we inferred that the prognosis of patients was not dependent on the overall proportion of immune infiltrating cells, but only correlated with certain types of cells.

### Dendritic cells were associated with prognosis

**Figure 3:**
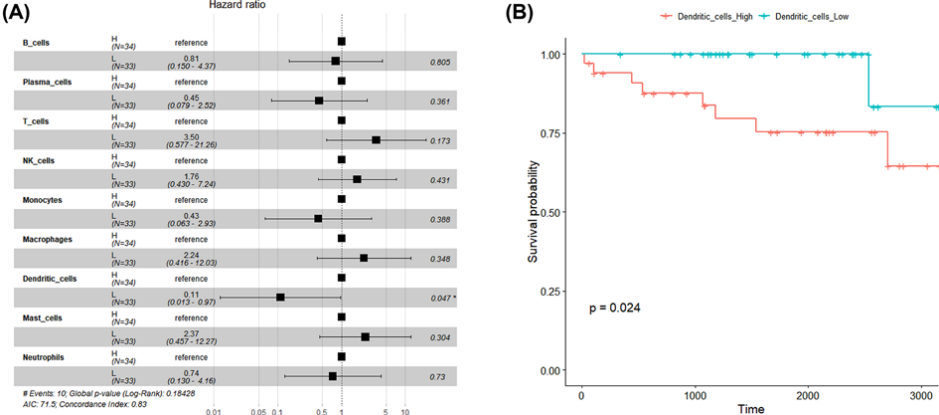
Content of dendritic cells affected prognosis (**A**) Hazard ratios in 67 samples from GEO dataset by CIBERSORT *P-*value. Boxes represented hazard ratios, and horizontal lines were 95% confidence intervals. (**B**) The survival curve of dendritic cells (*P* = 0.024).

For 67 samples with survival information, we first combined the 22 types of immune cells calculated by CIBERSORT into 10 types of immune cells by type. Then we used the survival package in R software, because the relative content of Eosinophils cells in more than 94% of the samples was 0, the relative content of 9 types of immune infiltrating cells except Eosinophils cells into high and low groups (higher than the median h, lower than the median L) according to the median to establish Cox regression model, as shown in Figure 3A. In the figure, HR hazard ratio was the most important conceptual risk ratio of Cox model. A value greater than 1 indicated that the factor had a higher risk of death than the reference and a value less than 1 indicated a lower death risk (lower 0.95 and upper 0.95 were 95% confidence intervals). The threshold value of *P*-value < 0.05 was used for screening. It could be seen from the figure that dendritic cells were significantly related to prognosis and survival, and a lower proportion of dendritic cells had a smaller risk of death. Figure 3B showed the corresponding survival curve.

### Enrichment analysis of GSEA

**Figure 4:**
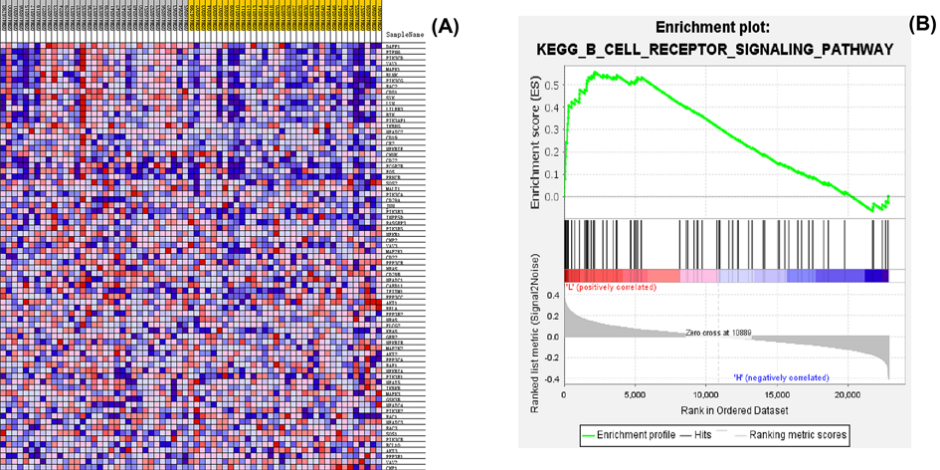
Gene set enrichment analysis (GSEA) (**A**) Heatmap of the relationship between a gene and a sample among all meningiomas samples. (**B**) B-cell receptor signaling pathway was significantly activated in the L group marked with dendritic cells.

In order to explore which pathways or functions were significantly activated or inhibited among the samples with different contents of dendritic cells, so as to affect the prognosis and survival, we used the GSEA 3.0 software to perform a gene set enrichment analysis. According to the median content of dendritic cells, the samples were divided into two groups: H group (High) with higher median and L group (Low) with lower median. All the genes in the samples were selected for analysis and the full results were provided in Supplementary Table S1. In addition, we illustrated a representative pathway that significantly activated in H group, i.e. B-cell receptor signaling pathway, in Figure 4. Figure 4A was a heatmap of the relationship between a gene and a sample. The horizontal axis was the sample grouping: gray was L group, and yellow was H group. The vertical axis was the gene in this pathway: the red blocks were strong correlated, and the blue blocks were weakly correlated. In Figure 4B, a vertical line in the middle of the figure represented a gene, the red below the vertical line represented L group, blue represented H group, and the *y*-axis represented the ES value of the enrichment score. It could be observed that the ES value was positive. It indicated that the B cell receptor signaling pathway was significantly activated in the L group marked with dendritic cells.

### Patterns of immune infiltration were correlated with survival

**Figure 5:**
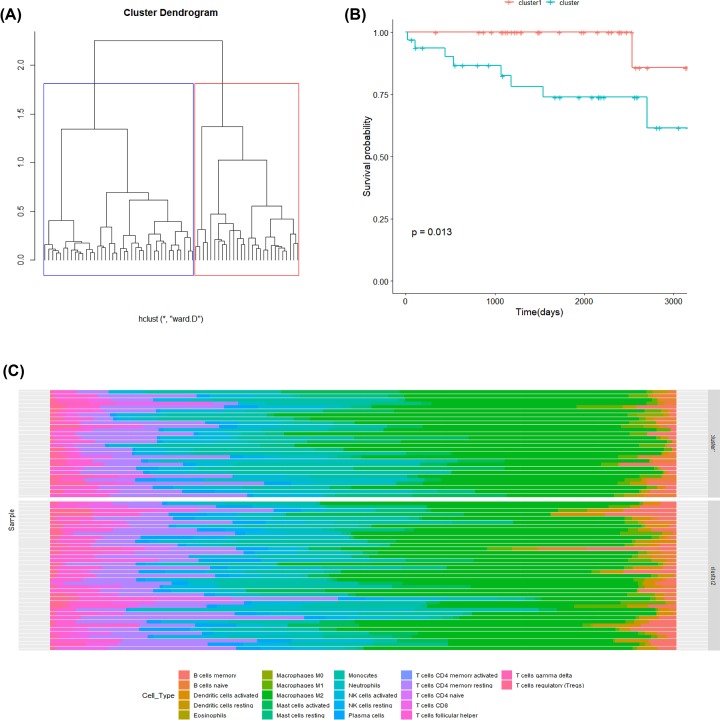
The patterns of immune infiltration and prognosis (**A**) The samples were clustered by Euclidean distance algorithm. (**B**) The survival curves of two clusters (*P* = 0.013). (**C**) The composition of tumor-infiltrating immune cells of two clusters.

According to the results of the previous study, we knew that the changes of some immune infiltrating cells affect the prognosis and survival to some extent. Therefore, we would like to know if we could distinguish different immune infiltration patterns through hierarchical clustering, and the relationship between each pattern and survival. First of all, we used the method of sum of squared errors (wss) to determine the optimal number of cluster classifications as two classes. Then, the Euclidean distance among all the samples calculated by their relative proportions of the 22 TIICs was used to classify the samples according to the determined number of classifications. Visualization of the sample clustering tree was carried out by R software. The different cluster classifications were represented by blue lines and red lines, and the results were shown in Figure 5A. Then we find that different clusters also have significantly different prognosis survival periods (*P-*value = 0.013), and the results are shown in Figure 5B. From Figure 5C, we could see that different clusters had significantly different compositions of immune infiltrating cell: the contents of B cells and dendritic cells in cluster 2 were higher than that in cluster 1, which was also confirmed from the above results of survival analysis and GSEA analysis.

## Discussion

Apart from malignant tumor cells, tumor tissues also include endothelial cells, fibroblasts, immune cells, and a large amount of growth factors, chemokines and cytokines [26,27]. Those components and their complicated interaction form the tumor-related microenvironment, which could obviously effects on the growth, invasion and metastasis of aggressive malignant cell [28,29]. During the development of malignant tumor cells, multiple immune cells of the host are recruited to and activated in the tumor microenvironment [30]. It is generally known that there is a complicated biological process that has prominent prognostic relevance between tumor cells and immune cells with both promoting and inhibiting roles of tumor [31]. Tumor-infiltrating immune cells are influential members of host immune responses to tumor, which also represent the characteristics of tumor immune microenvironment [32]. Despite tumor-infiltrating immune cells have been widely investigated in many cancers, such as breast cancer [33], renal cancer [34], gastric cancer [35], ovarian cancer [36] and pancreatic cancer [37], the composition, proportion and particular function of tumor-infiltrating immune cells are rarely reported in meningiomas to date. In the present study, we comprehensively explored the tumor-infiltrating immune cells in meningiomas samples based on the gene expression profiles with CIBERSORT algorithm.

We could see the proportion of different immune cells in the samples from the GEO dataset. It could be observed that these samples contained more M2 macrophages cells, reaching more than 36%. Macrophages are functionally and phenotypically characterized by the heterogeneity in distinct resident populations [38]. Researched showed that M2 macrophages were regarded as tumor-associated macrophages, which could bring to cancer invasion and metastasis [39,40]. Therefore, we inferred that M2 macrophages could provide a searchable target for the study of the occurrence and development of meningiomas. In addition, the composition of immune cells was very different in 68 meningiomas samples. We speculated that the change in the proportion of tumor-infiltrating immune cells might be an inherent feature, which could characterize individual and disease differences.

We also explored that the strong relationship between different *P*-value thresholds and cytolytic activity from GEO dataset. Previous research revealed that the overall proportion of 22 immune cells, as well as the proportion of single cell subdivision, was obviously related to prognosis survival in multiple other cancers, such as breast cancer [41,42]. The relationship between cytolytic activity and the *P-*value indicated that the *P-*value reflected the relative proportion of a sample composed of immune cells. Clinical outcome in patients with tumors is closed correlated with pattern of immune infiltration depending on the context and composition of immune cells [43,44]. The result of HR showed that the content of dendritic cells had a major effect on the prognosis. Dendritic cells are widespread recognized for the role in killing tumor [45]. However, dendritic cells have not been reported to be related to prognosis in meningiomas before. We elucidated their prognostic values in meningiomas for the first time.

GSEA analysis displayed that the B-cell receptor signaling pathway was significantly activated in the L group marked with dendritic cells. Therefore, we speculated that the content of dendritic cells might indirectly affect the content of B cells in the tumor microenvironment and thus affect the prognosis and survival of meningiomas. The study found that B-cell receptor signaling pathway could promote growth and survival of chronic lymphocytic leukemia [46,47]. Therefore, we speculated that the content of dendritic cells might indirectly affect the content of B cells in the tumor microenvironment and thus affect the prognosis and survival of meningiomas.

Furthermore, we carried out hierarchical clustering and identified two immune clusters. It was observed that two clusters of from GEO dataset presented different patterns of immune infiltration and prognosis by unsupervised clustering analysis. Recent research confirmed that different immune clusters had diverse prognosis in pan-cancer [48], which was consistent with our observation in meningiomas.

Briefly, our analysis of 22 immune cell subsets from GEO dataset displayed that different contents of dendritic cells significantly affected prognosis in meningiomas. B-cell receptor signaling pathway was significantly activated in the L group marked with dendritic cells. Moreover, different immune clusters had distinct survival outcomes. These findings suggested that certain tumor-infiltrating immune cells as well as immune infiltration patterns were relevant to the prognosis, which could be used as a potential therapeutic target for the diagnosis and treatment of meningiomas.

## Supplementary Material

Supplementary Table S1

## Abbreviations

GSEA: Gene set enrichment analysis
KEGG: Kyoto Encyclopedia of Genes and Genomes
