# Supplementary material for: Profiles of immune infiltration and its relevance to survival outcome in meningiomas
Source: Biosci Rep. 2020 May 14;40(5):BSR20200538. doi: 10.1042/BSR20200538 (PMC7225412; doi:10.1042/BSR20200538)
Supplement: Supplementary Table S1 [file BSR-2020-0538_supp.pdf]

Table S1 Significantly enriched pathways in GSEA of stratified samples by median content of dendritic cell.

NAME	SIZE	NES	NOM p-val
KEGG_TYPE_II_DIABETES_MELLITUS	46	1.728561	0.006493507
KEGG_CYTOSOLIC_DNA_SENSING_PATHWAY	51	1.811853	0.007843138
KEGG_LEISHMANIA_INFECTION	69	1.816799	0.0125
KEGG_VIRAL_MYOCARDITIS	66	1.805878	0.017094018
KEGG_AUTOIMMUNE_THYROID_DISEASE	47	1.731312	0.021097047
KEGG_TOLL_LIKE_RECEPTOR_SIGNALING_PATHWAY	99	1.643356	0.021141648
KEGG_HEMATOPOIETIC_CELL_LINEAGE	85	1.552251	0.021141648
KEGG_INTESTINAL_IMMUNE_NETWORK_FOR_IGA_PRODUCTION	44	1.71538	0.021598272
KEGG_GRAFT_VERSUS_HOST_DISEASE	37	1.708017	0.022869023
KEGG_NOD_LIKE_RECEPTOR_SIGNALING_PATHWAY	61	1.611335	0.026639344
KEGG_ASTHMA	27	1.756326	0.029166667
KEGG_ALLOGRAFT_REJECTION	34	1.703801	0.029661017
KEGG_CHEMOKINE_SIGNALING_PATHWAY	181	1.519306	0.03125
KEGG_ANTIGEN_PROCESSING_AND_PRESENTATION	79	1.635143	0.031712472
KEGG_REGULATION_OF_ACTIN_CYTOSKELETON	209	1.418368	0.03177966
KEGG_CELL_ADHESION_MOLECULES_CAMS	127	1.497074	0.035196688
KEGG_TYPE_I_DIABETES_MELLITUS	40	1.735205	0.03742204
KEGG_PRION_DISEASES	35	1.609831	0.037848607
KEGG_B_CELL_RECEPTOR_SIGNALING_PATHWAY	75	1.559609	0.042283297
KEGG_LEUKOCYTE_TRANSENDOTHELIAL_MIGRATION	114	1.485385	0.043933053
KEGG_STEROID_HORMONE_BIOSYNTHESIS	44	-1.4526	0.04483431
KEGG_JAK_STAT_SIGNALING_PATHWAY	152	1.482351	0.045643155
